# Admission Lower Serum Phosphate Ion Levels Predict Acute Hydrocephalus of Aneurysmal Subarachnoid Hemorrhage

**DOI:** 10.3389/fneur.2021.759963

**Published:** 2022-01-06

**Authors:** Yibin Zhang, Shufa Zheng, Haojie Wang, Guogong Chen, Chunwang Li, Yuanxiang Lin, Peisen Yao, Dezhi Kang

**Affiliations:** ^1^Department of Neurosurgery, Neurosurgery Research Institute, The First Affiliated Hospital, Fujian Medical University, Fuzhou, China; ^2^Fujian Key Laboratory of Precision Medicine for Cancer, The First Affiliated Hospital, Fujian Medical University, Fuzhou, China; ^3^Key Laboratory of Radiation Biology of Fujian Higher Education Institutions, The First Affiliated Hospital, Fujian Medical University, Fuzhou, China; ^4^Clinical Research and Translation Center, The First Affiliated Hospital, Fujian Medical University, Fuzhou, China; ^5^Fujian Clinical Research Center for Neurological Disease, The First Affiliated Hospital, Fujian Medical University, Fuzhou, China

**Keywords:** subarachnoid hemorrhage, aneurysm, hydrocephalus, risk factor, phosphate

## Abstract

**Introduction:** The relationship between serum phosphate ion (sPi) and the occurrence of acute hydrocephalus (aHCP) in aneurysmal subarachnoid hemorrhage (aSAH) remains largely unknown and controversial. The primary aim of this study was to investigate the association between sPi on admission and aHCP following aSAH.

**Methods:** The study included 635 patients over the age of 19 years diagnosed with aSAH in our institution from September 2012 to June 2018. Data on clinical characteristics, laboratory parameters, treatments, and outcomes were collected and analyzed. The association between lower sPi levels and aHCP was assessed in univariate and multivariate analyses. Propensity-score matching (PSM) analysis was performed to reduce significant differences in baseline characteristics between the aHCP group and non-HCP group.

**Results:** The overall incidence of aHCP following aSAH was 19.37% (123/512). Lower sPi levels were detected in patients with aHCP compared with those without [0.86 (0.67–1.06) vs. 1.04 (0.84–1.21) mmol/L] in the univariate analysis. In the multivariate analysis, lower sPi level, high modified Fisher (mFisher) grade, and high Hunt-Hess grade were associated with aHCP [odds ratios (OR) 1.729, 95% confidence interval (CI) 1.139–2.623, *p* = 0.01; mFisher OR 0.097,95% CI 0.055–0.172, *p* < 0.001; Hunt-Hess, OR 0.555, 95% CI 0.320–0.961, *P* = 0.036]. After PSM, the matched aHCP group had a significantly lower sPi level than the matched non-aHCP group [0.86 (0.67–1.06) vs. 0.94 (0.76–1.12) mmol/L, *p* = 0.044]. The area under the curve (AUC) of the sPi level and the logistic regression model based on these predictors (sPi, Hunt-Hess grade, and mFisher grade) was 0.667 and 0.840 (sensitivity of 88.6% and specificity of 68.4%) for predicting aHCP, respectively.

**Conclusions:** Lower sPi levels predict the occurrence of aHCP, and the model constructed by sPi levels, Hunt-Hess grade, and mFisher grade markedly enhances the prediction of aHCP after aSAH.

## Introduction

Aneurysmal subarachnoid hemorrhage (aSAH) is a fast developing, devastating, and life-threatening hemorrhagic stroke with high mortality and disability (1–3). Hydrocephalus (HCP) is a frequent complication of aSAH, with a reported incidence ranging from 6.5 to 85% (4–7), which is classified as acute (0–3 days post-hemorrhage), subacute (4–13 days post-hemorrhage), or chronic (14 days post-hemorrhage) (8, 9). Acute hydrocephalus (aHCP) is a potentially treatable cause of early neurological deterioration, and 20% of aSAH patients develop aHCP within 72 h (6, 8).

Numerous factors, including female sex, elderly age, higher mFisher grade, intraventricular hemorrhage, and laboratory findings, have been reported in prior literature to predict the occurrence of aHCP following aSAH (1, 7, 8, 10, 11). In recent years, considerable efforts have been focused on studying sensitive biomarkers for predicting aHCP (12–14), but reports of these biomarkers on the risk factors for aHCP development are inconsistent and controversial (13–16). Clinically, laboratory biomarkers have received extensive attention and research due to their convenience, practicality, and sensitivity (17). Nonetheless, additional laboratory testing of cerebrospinal fluid (CSF) is ubiquitous in most basic research based on biomarkers, which may impose additional risk and expense (17). Hence, it is necessary to explore inexpensive and convenient biomarkers to provide valuable information for appropriate clinical treatment in patients with aSAH and prediction of HCP or prognosis.

During the early development of HCP, the systemic inflammatory response is implicated *in vivo* in a range of physiological and pathological processes contributing to aHCP (12). Identifying high-risk aHCP patients in the early stage of aSAH is sufficiently important to provide timely appropriate interventions. The feasibility of using biomarkers to identify these high-risk patients with aHCP remains uncertain (12). Phosphate is involved in numerous physiological and pathophysiological processes *in vivo* via direct or indirect pathways, such as enzymes activity and energy metabolism (18, 19). Acute diseases can affect the accurate regulation of phosphate metabolism in the body (18). Various studies have shown serum phosphate levels are highly correlated with the occurrence and prognosis of acute cerebrovascular disease (20–23). Hypophosphatemia is a common treatable problem in the intensive care unit (ICU) and manifests many pathophysiological processes that occur in critical illness, including hemorrhagic stroke and other neurocritical diseases (24, 25). Weber et al. reported for the first time that profound changes in serum phosphate ion (sPi) might be related to reversible brain injury (21). Subsequently, impressive literature has reported that hypophosphatemia is associated with a disturbance of consciousness after spontaneous intracerebral hemorrhage (ICH), but its mechanism is still poorly understood (22). Emerging literature indicated that hypophosphatemia is associated with aHCP after ICH (20). However, the patients enrolled in this study were mixed with other causes of ICH, and the number of patients with ICH was small.

To the best of our understanding, no previous studies on aSAH have evaluated the effect of sPi level on aHCP after aSAH. We sought to investigate whether sPi level was linked to the severity of aSAH or other factors, such as sex, age, and blood pressure. Therefore, this study was performed to determine whether sPi level was associated with aHCP in aSAH patients.

## Materials and Methods

### Study Population

**Figure 1:**
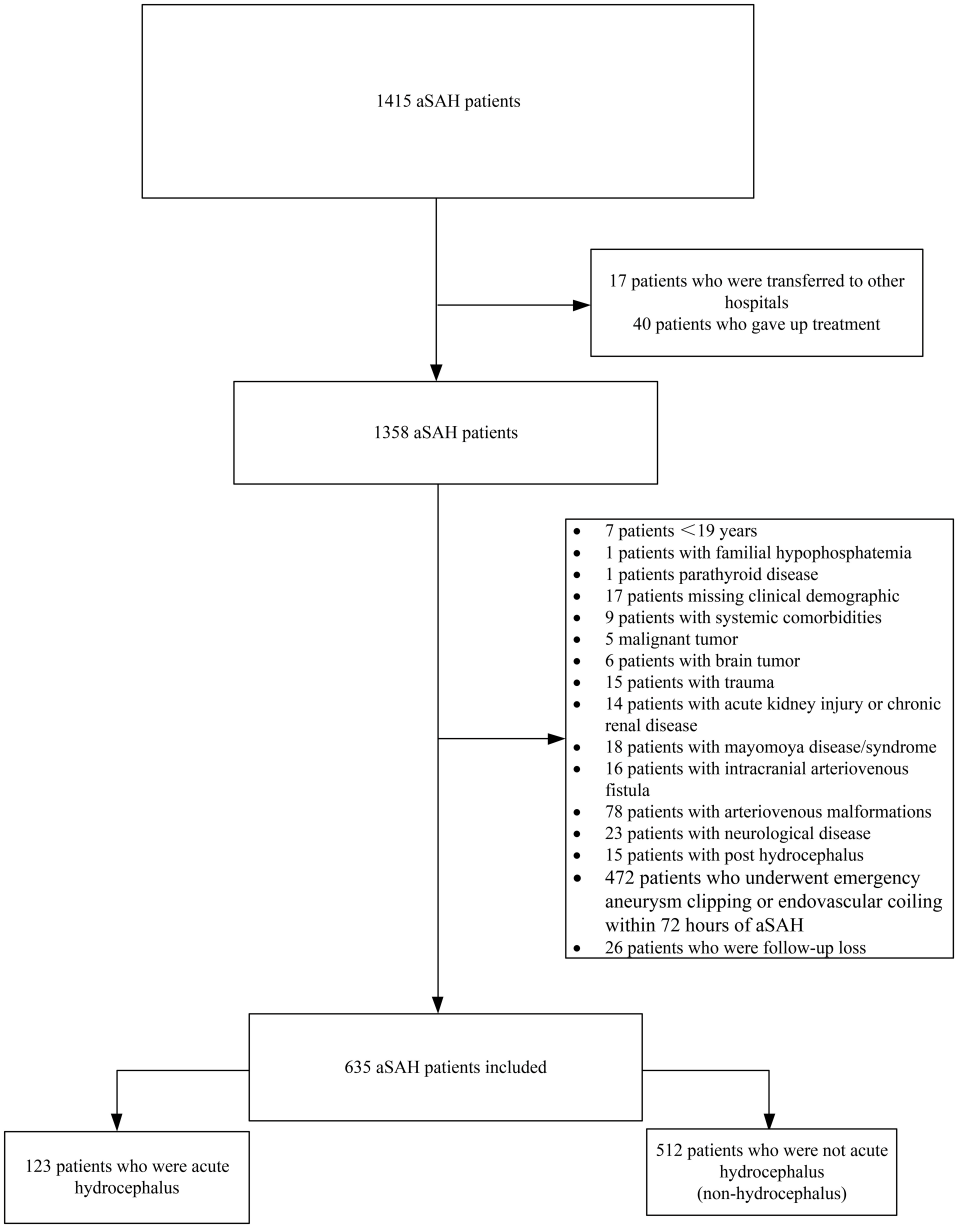
Study population of flow chart. aSAH, aneurysmal subarachnoid hemorrhage.

This was a single-center, retrospective observational study of aSAH patients admitted to the neurosurgery department in the First Affiliated Hospital of Fujian Medical University from September 2012 to June 2018. The study protocol was reviewed and approved by the hospital's institutional review board. Patients were included in the analysis with the following inclusion criteria: (1) age ≥19 years old; (2) Computed tomography (CT) scans confirmed the diagnosis of SAH on admission; (3) Computed tomography angiography (CTA) and/or digital subtraction angiography (DSA) diagnosed subarachnoid hemorrhage due to a ruptured cerebral aneurysm; (4) survival for no <72 h after aSAH to allow assessment of the development and prognosis of hydrocephalus. While exclusion criteria were listed as following: (1) age of <19 years; (2) a history of neurological disease including cerebrovascular disease, hemorrhagic or ischemic stroke and trauma; (3) patients presented with post hydrocephalus; (4) a history of familial hypophosphatemia or parathyroid disease; (5) patients underwent emergency aneurysm clipping or interventional embolization within 72 h after aSAH; (6)acute kidney injury or chronic kidney disease;7)concurrent systemic comorbidities. The flowchart of this study is present in Figure 1.

Clinical management followed the American Heart Association and American Stroke Association guidelines (7).

### Baseline Data Collection

Peripheral venous blood samples were obtained at admission within 30 min, and they were immediately sent to the clinical laboratory center. The biochemical analyses were completed within a 4-h period after samples were received. Clinical data and demographic information, including age, sex, medical history, time from onset of illness to admission, Hunt-Hess grade, mFisher grade, aneurysm location, and laboratory data, were collected. Laboratory data on admission included serum potassium, sodium, calcium and phosphate. aSAH patients were divided into mild aSAH (mFisher grade 0–2) or severe aSAH (mFisher grade 3–4) according to initial CT on admission and also classified into mild clinical conditions (Hunt-Hess grade I–III) or severe clinical conditions (Hunt-Hess grade IV–V) (26). mFisher score was dichotomized as “low grade (Grade 0, 1, 2)” and “high grade (Grade 3, 4),” Hunt-Hess grade as “low grade (Grade I, II, III)” and “high grade (Grade IV–V).” Aneurysms were classified into aneurysms and single aneurysms. “Multiple” denotes patients with two or more aneurysms, and “Single” denoted patients with only one aneurysm. Single aneurysm location was categorized into anterior cerebral artery (ACA), anterior communicating artery (ACoA), internal carotid artery (ICA), middle cerebral artery (MCA), posterior communicating artery (PCoA), and other locations.

### Diagnosis and Treatment of Hydrocephalus

The onset of hydrocephalus is rapid and persistent, occurring within 6 h after aSAH and remaining for at least 72 h. Therefore, aHCP was defined as the development of cerebral ventricle enlargement within 72 h of subarachnoid hemorrhage after aneurysmal rupture (5). The bicaudate index and relative bicaudate index were used to diagnose hydrocephalus (10). Two neurosurgeons and two radiologists independently evaluated the diagnostic imaging findings of acute hydrocephalus, and the consensus made the final judgment of more than three people. A following-up CT was performed depending on the patient's clinical condition. Emergency brain CT scans were performed in the presence of clinical deterioration, new focal neurological deficits, seizures, or gradual loss of consciousness (including rebleeding or aHCP) (5). If the patient had severe aHCP or progressive deterioration of consciousness or periventricular edema, immediate external ventricular drainage was performed (3).

### Outcome Assessment

All patients were followed up until 90 days after onset of aSAH or until death, depending on which happened first. Functional outcome was assessed 90 days after the aSAH onset using the modified Rankin scale (mRS) through follow-up, which was dichotomized into a favorable prognosis (mRS <3) and an unfavorable (mRS 3–6) prognosis (27). Follow-up information was gathered by outpatient reviews, telephone, or WeChat interviews. mRS scores range from 0 (no symptoms) to 6 (death) (28). Clinical outcome was evaluated by trained personals who were blinded to the subject's identity or clinical information.

### Statistical Analysis

All statistical analyses were conducted by SPSS software (version 25.0, IBM SPSS, IBM Corp), Prism 8.3.0 (GraphPad Software, San Diego, CA, USA), and MedCalc version 20.0.4 (MedCalc Software, Ostend, Belgium). For comparison of different groups for categorical variables, the χ2 test or Fisher's exact test was utilized. Normally distributed continuous variables are presented as means ± standard deviation (SD) employing Student *t*-test, and non-normally distributed variables as median (interquartile range, IQR) using the Mann-Whitney U test or Kruskal–Wallis test intergroup differences. Median with IQR is shown for all scatter plots. Spearman's correlation analysis was performed to access the correlation coefficient between two variables. All clinical variables associated with aHCP at *P*<*0.10* in the univariate analysis were considered for entry into the multivariate models. In the multivariate model, sPi level was dichotomized as “≤optimal cutoff value” and “> optimal cutoff value.” Multivariable logistic regression models were used to develop the combined models. The receiver operating characteristic (ROC) curve was constructed, and the area under the AUC was calculated to access the predictive power of the sPi for aHCP. Also, a combined model (constructed by multivariable logistic regression) ROC analysis was undertaken to potentially identify a more potent predictive hydrocephalus model. PSM analysis was conducted to further account for significant differences in baseline characteristics between the two groups. The covariates significantly associated with aHCP by univariate were included in the matching. The nearest neighbor matching algorithm was performed to match the aHCP group and the non-aHCP group at a ratio of 1:1. *P*<*0.05* was accepted as statistically significant.

## Results

### Patient Characteristics

Six hundred thirty-five aSAH patients were enrolled in the study based on the inclusion criteria between September 2012 and June 2018. The mean age was (54.26 ± 10.77) years, and 400 (63%) participants were female. Ninety-six patients (15.1%) were in severe clinical condition on admission (Hunt-Hess grade IV–V). Two hundred fifty-nine patients (40.8%) had severe aSAH (mFisher score 3–4) on admission CT. The concentrations of serum potassium, sodium, calcium and sPi at hospital admission were [3.94 (3.65–4.22) mmol/L, 140.80 (138.0–143.0) mmol/L, 2.17 (2.08–2.26) mmol/L, 1.01 (0.82–1.19) mmol/L], respectively. Aneurysms were treated with surgical clipping in 436 (68.7%) patients, while the other 199 (31.3%) received endovascular coiling. All enrolled participants were segregated into the aHCP group (*n* = 512) and non-aHCP group (*n* = 123). The overall incidence of aHCP after aSAH was 19.37% (123/512).

### Association of sPi Levels With aHCP

**Figure 2:**
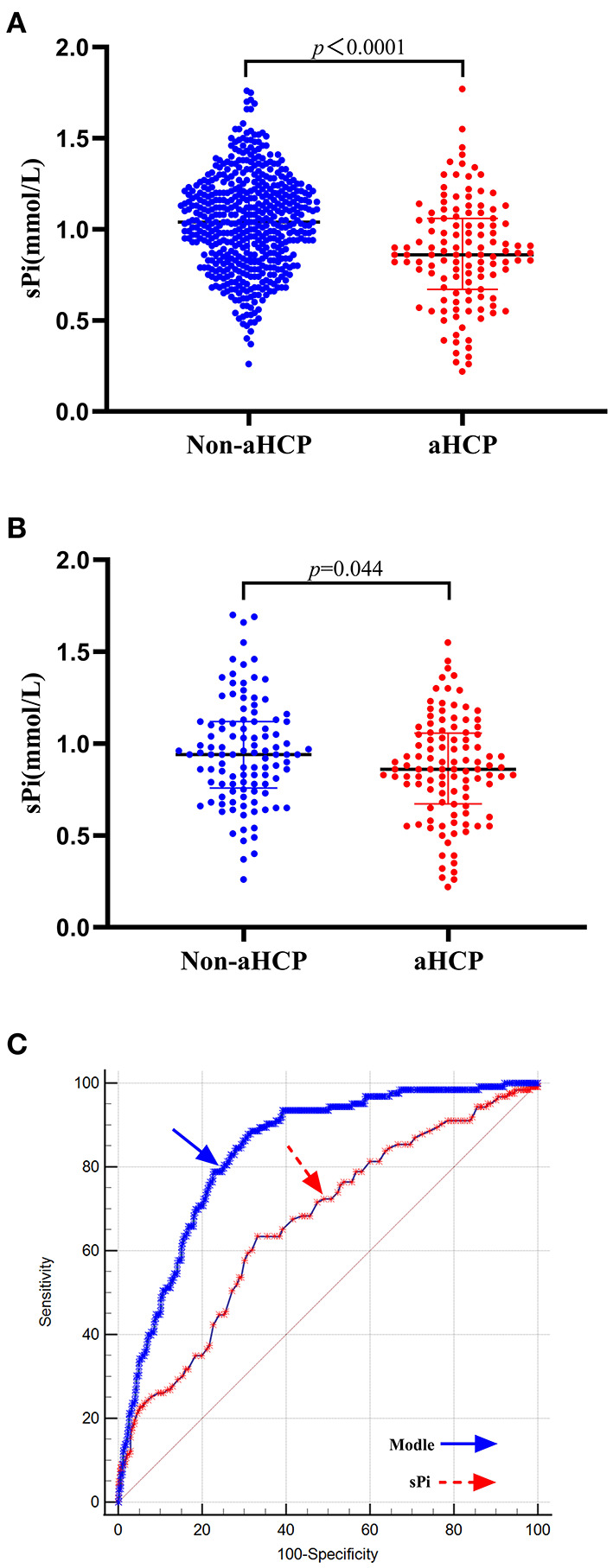
Association of sPi levels with aHCP. **(A)** sPi levels in patients with non-aHCP (*n* = 512) and aHCP (*n* = 123). **(B)** sPi levels in patients with non-aHCP (*n* = 108) and aHCP (*n* = 108) after PSM. **(C)** ROC curve analysis for predicting aHCP. The optimal cutoff value for sPi level as a predictor for aHCP in aSAH patients was determined to be 0.93 mmol/L (AUC was 0.667, the sensitivity was 63.4%, and the specificity was specificity 66.8%). The AUC of the logistic regression model based on these predictors (sPi, Hunt-Hess grade, and mFisher grade) was 0.840 with a sensitivity of 88.6% and specificity of 68.4% for aHCP, which is a stronger aHCP predictor than a single sPi level. Median with IQR was shown for all scatter plots in **(A,B)**. Mann-Whitney tests were performed to compare differences between groups. aSAH, aneurysmal subarachnoid hemorrhage; aHCP, acute hydrocephalus; AUC, area under the curve; IQR, interquartile range; PSM, Propensity-score matching; ROC, Receiver operating curve; sPi, serum phosphate ion.

Demographic characteristics, clinical features, and laboratory data with potential significance for aHCP are summarized in Table 1. On univariate analysis, factors associated with the occurrence of aHCP following aSAH were hypertension (*p* < 0.001), diabetes mellitus (*p* = 0.007), systolic blood pressure (SBP) (*p* < 0.001), diastolic blood pressure (DBP) (*p* = 0.021), admission Hunt-Hess grade (*p* < 0.001), mFisher grade (*p* < 0.001), serum potassium (*p* = 0.004), serum calcium (*p* = 0.042) and sPi (*p* < 0.001). Lower sPi levels were detected in patients with aHCP compared with those without [0.86 (0.67–1.06) mmol/L vs. 1.04 (0.84–1.21) mmol/L, *p* < 0.001] (Table 1; Figure 2A). Multivariable analysis revealed that lower sPi level, higher mFisher grade, and higher Hunt-Hess grade were associated with aHCP [Table 1; sPi, odds ratios (OR) 1.729, 95% confidence interval (CI) 1.139–2.623, *p* = 0.01; mFisher OR 0.097,95%CI 0.055–0.172, *p* < 0.001; Hunt-Hess, OR 0.555, 95% CI 0.320–0.961, *p* = 0.036], while hypertension, diabetes mellitus, SBP, DBP, serum potassium and serum calcium were not associated with aHCP.

**Table 1:** Univariate and multivariate analyses of association with hydrocephalus following aSAH.

**Characteristics**	**Non-aHCP** **(*n* = 512)**	**aHCP** **(*n* = 123)**	**Univariate** ***P-*value**	**Multivariate analysis**		
				**OR**	**95%CI**	***P* value**
Age, mean ±SD, years	53.88 ± 10.92	55.85 ±9.97	0.055	1.007	0.984–1.031	0.560
Gender (N, %)			0.175			
Male	196 (38.3)	39 (31.7)				
Female	316 (61.7)	84 (68.3)				
**Medical history**						
Smoking (N, %)	87 (17.0)	16 (13.0)	0.282			
Alcohol (N, %)	56 (10.9)	11 (8.9)	0.518			
Hypertension (N, %)	288 (56.3)	47 (38.2)	<0.001	0.838	0.489–1.435	0.518
Diabetes mellitus (N, %)	45 (8.8)	21 (17.1)	0.007	0.757	0.390–1.472	0.412
Hyperlipidemia (N, %)	84 (16.4)	24 (19.5)	0.410			
**Admission vital signs**						
SBP, mean ±SD, mmHg	139.99 ± 24.40	150.14 ± 27.62	<0.001	1.005	0.992–1.017	0.445
DBP, mean ±SD, mmHg	83.74 ± 13.77	87.15 ± 14.80	0.021	0.995	0.974–1.017	0.672
**Hunt-Hess grade (N, %)**			<0.001	0.555	0.320–0.961	0.036
Grade I–III	460 (89.8)	79 (64.2)				
Grade IV, V	52 (10.2)	44 (35.8)				
**mFisher grade (N, %)**			<0.001	0.097	0.055-0.172	<0.001
Grade 0–2	288 (56.3)	17 (13.8)				
Grade 3–4	224 (43.8)	106 (86.2)				
**Aneurysm characteristics**						
Multiple aneurysms (N, %)	67 (13.1)	15 (12.2)	0.791			
**Single aneurysms location (N, %)**			0.512			
ACA	25 (5.6)	7 (6.5)				
AcoA	125 (28.1)	31 (28.7)				
ICA	77 (17.3)	12 (11.1)				
MCA	90 (20.2)	21 (19.4)				
PcoA	101 (22.7)	32 (29.6)				
Others	27 (6.1)	5 (4.6)				
Time from onset to admission, IQR, h	14 (10–17)	15 (6–17)	0.354			
**Admission laboratory**						
Serum potassium, IQR,mmol/L	3.97 (3.67–4.24)	3.83 (3.45–4.12)	0.004	1.006	0.609–1.662	0.980
Serum sodium, IQR, mmol/L	140.8 (138.33–142.88)	141.0 (137.70–143)	0.906			
Serum calcium, IQR, mmol/L	2.18 (2.08–2.27)	2.15 (2.08–2.24)	0.042	0.830	0.145–4.756	0.835
sPi, IQR, mmol/L,	1.04 (0.84–1.21)	0.86 (0.67–1.06)	<0.001	1.729	1.139–2.623	0.01
**Treatment (N, %)**			0.156			
Clipping	345 (67.4)	91 (74.0)				
Coiling	167 (32.6)	32 (26.0)				

*ACA, anterior cerebral artery; ACoA, anterior communicating artery; aSAH, aneurysmal subarachnoid hemorrhage; aHCP, acute hydrocephalus; CI, confidence interval; DBP, diastolic blood pressure; ICA, internal carotid artery; IQR, interquartile range; MCA, middle cerebral artery; mFisher, modified Fisher; OR, odds ratios; PcoA, posterior communicating artery; sPi, serum phosphate ion; SD, standard deviation; SBP, systolic blood pressure*.

After PSM, no significant differences were observed in hypertension, diabetes mellitus, SBP, DBP, serum potassium and serum calcium, admission Hunt-Hess grade, mFisher grade between HCP and non-HCP groups. The matched HCP group had a significantly lower sPi level than the matched non-HCP [0.86 (0.67–1.06) vs. 0.94 (0.76–1.12) mmol/L, *p* = 0.020; Table 2; Figure 2B].

**Table 2:** Univariate analyses of association with hydrocephalus following aSAH after PSM.

**Characteristics**	**Non-aHCP** **(*n* = 108)**	**aHCP** **(*n* = 108)**	**Univariate** ***P-*value**
Age, mean ±SD, years	55.83 ± 11.24	55.27 ± 9.72	0.693
**Gender (N, %)**			0.254
Male	42 (38.9)	34 (31.5)	
Female	66 (61.1)	74 (68.5)	
**Medical history**			
Smoking (N, %)	17 (15.7)	15 (13.9)	0.702
Alcohol (N, %)	10 (9.30)	11 (10.2)	0.818
Hypertension (N, %)	48 (44.4)	45 (41.7)	0.680
Diabetes mellitus (N, %)	13 (12.0)	19 (17.6)	0.250
Hyperlipidemia (N, %)	19 (17.6)	21 (19.4)	0.726
**Admission vital signs**			
SAP, mean ± SD, mmHg	149.09 ± 28.01	148.92 ± 27.45	0.963
DBP, mean ± SD, mmHg	85.13 ± 14.14	86.68 ± 15.34	0.442
**Hunt-Hess grade (N, %)**			0.134
Grade I-III	77 (71.3)	73 (61.9)	
Grade IV, V	31 (28.7)	35 (38.1)	
**mFisher grade (N, %)**			1.00
Grade 0-2	17 (15.7)	17 (15.7)	
Grade 3-4	91 (84.3)	91 (84.3)	
**Aneurysm characteristics**			
Multiple aneurysms (N,%)	18 (16.7)	14 (13.0)	0.444
**Single aneurysm location**			0.208
**(N, %)**			
ACA	8 (8.9)	6 (6.4)	
AcoA	32 (35.6)	28 (29.8)	
ICA	13 (14.4)	11 (11.7)	
MCA	20 (22.2)	16 (17.0)	
PcoA	13 (14.4)	29 (30.9)	
Others	4 (4.4)	4 (4.3)	
Time from onset to	13 (8–16.75)	15 (6–17)	0.987
admission, IQR, h			
**Admission laboratory**			
Serum potassium, IQR, mmol/L	3.85 (3.61–4.15)	3.86 (3.45–4.12)	0.995
Serum sodium, IQR, mmol/L	140.55 (138.0–142.10)	140.75(137.63–143.0)	0.790
Serum calcium, IQR, mmol/L	2.15 (2.05–2.25)	2.16(2.08–2.24)	0.443
sPi, IQR,mmol/L	0.94 (0.76–1.12)	0.86(0.67–1.06)	0.044
**Treatment (N, %)**			0.759
Clipping	78(72.2)	80(74.1)	
Coiling	30(27.8)	28(25.9)	

*ACA, anterior cerebral artery; ACoA, anterior communicating artery; aSAH, aneurysmal subarachnoid hemorrhage; aHCP, acute hydrocephalus; CI, confidence interval; DBP, diastolic blood pressure; ICA, internal carotid artery; IQR, interquartile range; MCA, middle cerebral artery; mFisher, modified Fisher; OR, odds ratios; PcoA, posterior communicating artery; PSM, propensity-scores matching; sPi, serum phosphate ion; SD, standard deviation; SBP, systolic blood pressure*.

ROC curve analysis demonstrated an AUC (area under the curve) of 0.667 (95% CI 0.629–0.704, *p* < 0.0001) (Sensitivity = 63.4%; Specificity = 66.8%), and the best cutoff value for sPi level as a predictor for aHCP in aSAH patients was determined as 0.93 mmol/L. The AUC of the logistic regression model based on these predictors (sPi, Hunt-Hess grade, and mFisher grade) was 0.840 (95% CI 0.809–0.868, *p* < 0.0001) with a sensitivity of 88.6% and specificity of 68.4% for aHCP, which is a stronger aHCP predictor than a single sPi level (AUC: 0.667) (Figure 2C).

### Correlation Between sPi Level and Initial Clinical Status at Admission

To Compare patients in terms of sPi concentration, aHCP rate, the incidence of poor prognosis, mFisher grade, Hunt-Hess grade, and SBP were significantly higher in patients with lower sPi (sPi ≤ 1.0 mmol/L) concentration than those with higher sPi concentration (sPi > 1.0 mmol/L). Moreover, serum potassium, sodium, and calcium were lower in patients with higher sPi concentrations (Table 3).

**Table 3:** Patients' demographics and baseline characteristics by sPi concentration.

**Characteristics**	**Lower serum Phosphorus** **(≤1.00mmol/L)**	**Higher serum Phosphorus** **(>1.00mmol/L)**	**Univariate** ***P-*value**
Number of patients	312	323	
aHCP (N,%)	84 (26.9)	39 (12.1)	<0.001
Age, mean ± SD, yrs	54.67 ± 10.38	53.86 ± 11.13	0.342
**Gender (N, %)**			0.083
Male	126 (40.4)	109 (33.7)	
Female	186 (59.6)	214 (66.3)	
**Medical history**			
Smoking (N, %)	54 (17.3)	49 (15.2)	0.465
Alcohol (N, %)	36 (11.5)	31 (9.6)	0.426
Hypertension (N, %)	157 (50.3)	178 (55.1)	0.227
Diabetes mellitus (N, %)	33 (10.6)	33 (10.2)	0.882
Hyperlipidemia (N, %)	48 (15.4)	60 (18.6)	0.285
**Admission vital signs**			
SAP, mean ± SD, mmHg	145.23 ± 26.74	138.79 ± 13.55	0.001
DBP, mean ± SD, mmHg	84.61 ± 14.78	84.20 ± 13.29	0.709
**Hunt-Hess grade (N, %)**			<0.001
Grade I-III	246 (78.8)	293 (90.7)	
Grade IV, V	66 (21.2)	30 (9.3)	
**mFisher grade (N, %)**			<0.001
Grade 0-2	146 (46.8)	230 (71.2)	
Grade 3-4	166 (53.2)	93 (28.8)	
**Aneurysm characteristics**			
Multiple aneurysms (N, %)	45 (14.4)	37 (11.5)	0.265
**Single aneurysms location (N,%)**			0.074
ACA	9 (3.4)	23 (8.0)	
AcoA	77 (28.8)	79 (27.6)	
ICA	36 (13.5)	53 (18.5)	
MCA	63 (23.6)	51 (17.8)	
PcoA	66 (24.7)	64 (22.4)	
Others	16 (6.0)	16 (5.6)	
Time from onset to admission, IQR, h	14.0 (8.0–18.0)	15.0 (10.0–17.0)	0.949
**Admission laboratory**			
Serum potassium, IQR, mmol/L	3.83 (3.56–4.10)	4.05 (3.74-4.33)	<0.001
Serum sodium, IQR, mmol/L	140.45 (137.60–142.80)	141.0 (139.0–143)	0.038
Serum calcium, IQR, mmol/L	2.13 (2.04–2.21)	2.22 (2.14–2.30)	<0.001
**Treatment (N, %)**			0.053
Clipping	226 (72.4)	211 (65.3)	
Coiling	86 (27.6)	112 (34.7)	
**mRS (N, %)**			0.015
0–2	259 (83)	290 (89.8)	
3–6	53 (17)	33 (10.2)	

*ACA, anterior cerebral artery; ACoA, anterior communicating artery; aSAH, aneurysmal subarachnoid hemorrhage; aHCP, acute hydrocephalus; CI, confidence interval; DBP, diastolic blood pressure; ICA, internal carotid artery; IQR, interquartile range; MCA, middle cerebral artery; mFisher, modified Fisher; mRS, modified Rankin Scale; OR, odds ratios; PcoA, posterior communicating artery; sPi, serum phosphate ion; SD, standard deviation; SBP, systolic blood pressure*.

**Figure 3:**
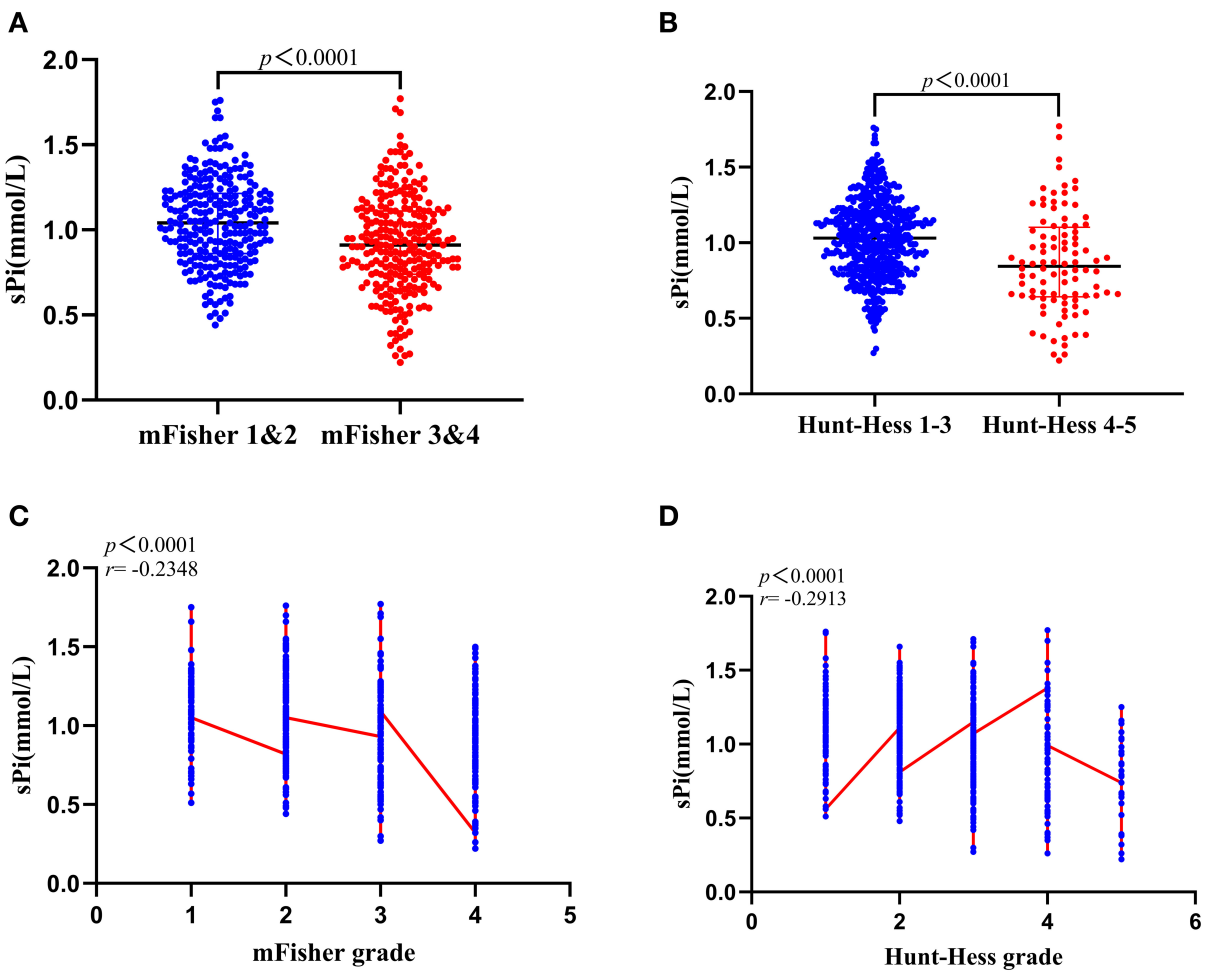
Correlation between sPi level and initial clinical status at admission. **(A)** sPi levels in patients with mild (mFisher 1&2, *n* = 265) and severe (mFisher 1&2, *n* = 259) radiologic status. **(B)** sPi levels in patients with mild (Hunt-Hess grade 1–3, *n* = 539) and severe (Hunt-Hess grade 4–5, *n* = 96) clinical conditions. **(C)** The correlation of sPi levels with mFisher grade (mFisher 0 was not imputed). **(D)** The correlation of sPi levels with Hunt-Hess grade. Median with IQR was shown for all scatter plots in **(A,B)**. Mann-Whitney tests were performed to compare differences between groups. Correlations were determined using Spearman's correlation analysis. aSAH: aneurysmal subarachnoid hemorrhage; IQR: interquartile range; sPi, serum phosphate ion; mFisher: modified Fisher.

The sPi level was significantly lower in aSAH patients with mFisher grade (3, 4) compared to mFisher (1, 2) [1.81 (1.49–2.28) vs. 1.42 (1.20–1.67) mmol/L, *p* < 0.0001; Figure 3A]. Furthermore, patients with Hunt-Hess (IV–V) had lower sPi concentration than those with Hunt-Hess (I–III) [0.85 (0.64–1.10) vs. 1.03 (0.84–1.20) mmol/L, *p* < 0.0001; Figure 3B]. Spearman analyses revealed a clear negative correlation between sPi and mFisher grade (*r* = −0.2348, *p* < 0.001; Figure 3C), between sPi and Hunt-Hess grade (*r* = −0.2913, *p* < 0.001; Figure 3D), respectively. Correlation analyses revealed a negative correlation between sPi and SBP (*r* = −0.137, *P* = 0.001). No correlation was detected between sPi and age or sex (*P*>0.05).

### Association of sPi Level With Unfavorable 90 Days Functional Outcome

**Figure 4:**
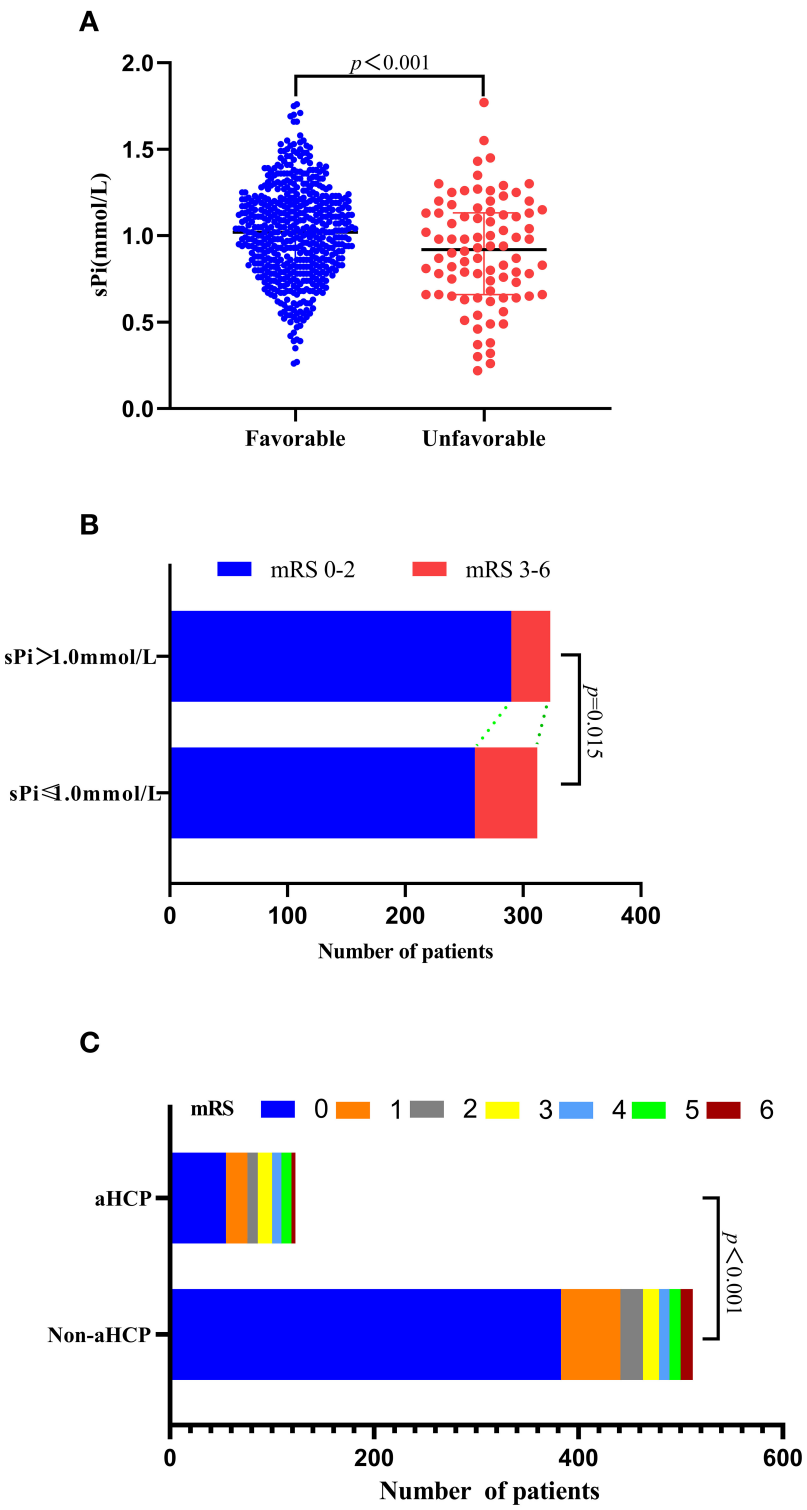
Association of sPi level with unfavorable 90 days functional outcome. **(A)** sPi levels in aSAH patients with favorable (mRS 0–2, *n* = 549) and unfavorable (mRS 3–6, *n* = 86) outcome. **(B)** Function outcome at 90 days for patients with lower sPi (sPi ≤ 1.0 mmol/L, *n* = 312) and higher sPi (sPi>1.0 mmol/L, *n* = 323) than medium level (1.0 mmol/L). Data are reported as scatter-dot plots and median with IQR in **(A)**. **(C)** Distribution of functional outcomes at 90 days between patients with aHCP and non-aHCP. Mann-Whitney tests were performed to compare differences in sPi levels between patients with favorable (mRS 0–2) and unfavorable outcomes (mRS 3–6). aSAH: aneurysmal subarachnoid hemorrhage; IQR, interquartile range; mRS: modified Rankin Scale; sPi, serum phosphate ion.

Compared with the favorable functional group, the sPi level at admission was markedly lower in the unfavorable functional group [0.92 (0.66–1.13) vs. 1.02 (0.83–1.20) mmol/L, *p* < 0.001, Figure 4A]. Patients with a sPi level below 1.0 mmol/L (the median level of sPi in all patients) have a worse prognosis than patients with a sPi level >1.0 mmol/L (Figure 4B). In addition, the incidence of poor prognosis in aSAH patients with aHCP was higher than those without HCP. Figure 4C detailed the distribution of mRS scores for the two groups of patients. According to the ROC curve, the AUC of sPi level was 0.602 (95% CI 0.563–0.640; *p* = 0.003) (Sensitivity = 25.58%; Specificity = 91.99%) for mRS based on a cut-off value of 0.66 mmol/L.

## Discussion

In the present study, we applied multivariate logistic regression analysis combined with PSM to access the association between sPi levels and aHCP. The significant findings of this study were as follows: (1) A lower admission sPi level was considered as a risk factor of aHCP; (2) mFisher scale and Hunt-Hess grade were negatively correlated with sPi, respectively; (3) Patients with severe aSAH (higher mFisher score and Hunt-Hess grade) have significantly lower sPi levels; (4) The sPi level was negatively associated with SBP; (5) The model constructed by sPi levels, Hunt-Hess grade, and mFisher scale markedly improves the prediction of aHCP after aSAH. Risk factors for aHCP have been reported, including mFisher score, Hunt-Hess grade in aSAH patients, which were balanced in this study. After PSM, no significant differences were observed in the mFisher score, Hunt-Hess grade between the two groups. In the univariate analysis, the matched aHCP group had a statistically significantly lower sPi level than the matched non-aHCP group and sPi was the only factor affecting aHCP. Therefore, the sPi level was implicated as a risk factor for aHCP, which was confirmed after PSM analysis. The model constructed by sPi levels, Hunt-Hess grade, and mFisher grade had an AUC of 0.840 with an associated sensitivity of 88.6% and specificity of 68.4% for predicting aHCP. Superior to the single sPi level or mFisher grade, the model demonstrated the best predictive power for aHCP, which should serve as a valuable tool to predict the occurrence of aHCP in aSAH patients. Previously reported literature demonstrated that aHCP and diffuse cerebral edema are more common in patients with non-traumatic intracranial hemorrhage accompanied by decreased sPi levels (20). Based on it, we listed aSAH from non-traumatic ICH to explore the relationship between decrease sPi levels and aHCP. The present study consolidated and expanded previous observations (20) and constructed an unexpected and novel combined model for predicting aHCP.

Phosphate metabolism is an evolving field of basic and clinical research. Phosphate is essential for all living cells, accounting for approximately 1% of human body mass. The most phosphate (85%) is found in bones and teeth, and the remaining 15% is distributed in body fluids and other cells (18, 19). As an energy source in the form of Adenosine Triphosphate (ATP), phosphate is crucial for cell metabolism and the normal development of bones. Phosphate is a vital mineral that participates in many physiological pathways and mineralization, the essential elements of membrane composition, such as the formation of various enzymes, the components of DNA and RNA, cell signaling, energy storage, transfer, and maintenance of acid-base equilibrium (19, 23, 25).

Decreased sPi concentration is a common clinical phenomenon after non-traumatic ICH, including aSAH (20, 25), which may be caused by increased renal excretion, decreased intestinal absorption, or internal redistribution of inorganic phosphate (19, 25). The redistribution of inorganic phosphate across cell membranes is the most common cause of hypophosphatemia in patients and can be caused by various clinical conditions (29). The underlying mechanism is attributed to the following reasons. Firstly, spontaneous hyperventilation is very common in patients with aSAH (30). In respiratory alkalosis, a decrease in carbon dioxide can trigger an increase in intracellular pH, which stimulates phosphofructokinase, leading to increased glycolysis and the incorporation of phosphate into organic intermediates (31, 32). Therefore, this causes a decrease in phosphate in the cell, which transfers phosphate into the cell. Some reports in the literature have revealed that during hyperventilation, the patient's serum phosphate level is as low as 0.3 mmol/L (30), which may be the most common cause of hypophosphatemia in hospitalized patients. Reduced sPi concentration caused by respiratory alkalosis is also seen in patients with head injuries, acute strokes, and mechanical ventilation (31, 33). Secondly, high serum levels of endogenous or exogenous catecholamines (such as epinephrine and norepinephrine) in patients after aSAH can lead to a decrease in serum phosphate (34). Reported literature hinted sPi is inversely related to the paroxysmal sympathetic storm and increased plasma epinephrine (35). Adrenaline can raise blood pressure, and our data also indicated that patients with lower sPi levels had higher SBP than those with higher sPi levels. Our findings are consistent with the literature in implying a negative correlation between the initial sPi concentration and plasma epinephrine (34, 36). Thirdly, serum phosphate levels are negatively correlated with the levels of inflammatory cytokines such as interleukin 6 (IL-6) and tumor necrosis factor-alpha (TNF-α) (10, 37). Elevated inflammatory factors lead to internal redistribution of phosphate, and decreased serum phosphate may be due to increased use of phosphate by immune cells.

aHCP is not an uncommon complication and is a potentially treatable cause of early neurological deterioration following aSAH. Systemic inflammatory response syndrome is common in aSAH (12, 38). aHCP is characterized by enlargement of the CSF-filled brain ventricles from failed CSF homeostasis. Since the 1840's, inflammation in the brain and the CSF spaces have been detected in both post-hemorrhagic and post-infectious hydrocephalus (39). Inflammatory cells might be an independent predictive factor associated with aHCP after aSAH. Recent studies have begun to reveal the molecular mechanisms by which inflammation-regulating cytokines, immune cells, and signaling pathways-contribute to the pathogenesis of hydrocephalus (10, 39). The cytokines, immune cells, and signal pathways all require the participation of phosphate. aSAH can accelerate cell apoptosis. Phosphate is released from dead cells into blood vessels, promotes vascular inflammation and endothelial cell apoptosis (39). In the present study, a reduction of serum phosphate was observed in patients with aHCP following aSAH, supporting a possible link between phosphate metabolism, inflammation, and hydrocephalus.

Numerous mechanisms have been implicated as causative factors for the development of aHCP following aSAH, including obstruction of the arachnoid granulations by blood products, alterations in CSF dynamics, and adhesions within the ventricular system (6, 10). Emerging data have established that inflammatory pathways are likely to play an essential role in the pathogenesis of aHCP. Inflammatory markers in CSF and peripheral blood are associated with the possibility of developing aHCP (9, 10, 12, 39). Lower sPi levels are negatively correlated with the levels of inflammatory cytokines (19). This study determined that a lower admission sPi level was considered a risk factor for aHCP. It seems consistent with previous literature that inflammation can induce aHCP (39).

Impaired early energy metabolism in the brain may be another mechanism that causes aHCP after aSAH. In this study, we observed decreased sPi concentration was closely related to the severity of aSAH, consistent with existing literature (20). Phosphate is the fundamental component of nucleic acids and phospholipids, and is one of the components of high-energy phosphate bonds, but also involved in protein phosphorylation, cell membrane composition, and the synthesis of various enzymes in the body. sPi is related to ATP production and 2,3-diphosphoglycerate (DPG) activity in red blood cells, which helps to release oxygen from hemoglobin (40). The low 2,3-DPG associated with decreased sPi concentration can cause the oxygen dissociation curve to shift to the left, red blood cell damage, hypoxia of tissue cells, and severe insufficiency of ATP energy supply, resulting in a decrease in oxygen release, thereby impairing brain energy metabolism, aggravating brain damage, and affecting the virtuous circulation of CSF (41). The above process may lead to subsequent aHCP. In addition, lower sPi levels may aggravate the destruction of the blood-brain barrier and further aggravate the occurrence of aHCP.

The influence of sPi levels on the prognosis of critically ill patients, including aSAH, is controversial (20, 25). Our current data demonstrated that lower sPi levels at admission were observed in patients with unfavorable outcomes. However, according to the ROC curve, the AUC of sPi is 0.602 for mRS, indicating that the predictive value of sPi in predicting poor prognosis is weak. The presence of a lower sPi level may be just another indicator of disease severity, not a major contributor factor to disease severity. This hypothesis is supported by our findings that there is a negative correlation between the initial phosphate concentration and the commonly used disease severity scores, including mFisher score and Hunt-Hess grade. This association has been observed in some previous studies, but the opposite results have also been reported (20). Their research objects are not patients with sICH. Therefore, it seems reasonable that the conclusions are different.

Limitations of this study include sample size and observational single-center study, which may limit the generality of our results. Furthermore, laboratory data collected under the clinical conditions of admission are initial values, and enhancing the analysis of changed review data may improve the accuracy and credibility of our study. Thirdly, factors affecting sPi levels have not been considered, including eating habits, food intake, and hormone levels. Finally, related inflammatory cytokines, such as *C* reactive protein, procalcitonin, and IL-6, were not included in the analysis.

## Conclusion

Lower sPi levels predict the occurrence of aHCP, and the model constructed by sPi levels, Hunt-Hess grade, and mFisher grade markedly enhances the prediction of aHCP after aSAH. Further multicenter randomized studies are needed to determine whether changes in sPi levels over time are related to the occurrence of aHCP.

## Data Availability Statement

The original contributions presented in the study are included in the article/supplementary material, further inquiries can be directed to the corresponding authors.

## Ethics Statement

The studies involving human participants were reviewed and approved by the Ethics Committee of the First Affiliated Hospital of Fujian Medical University. Written informed consent from the participants' legal guardian/next of kin was not required to participate in this study in accordance with the national legislation and the institutional requirements.

## Author Contributions

YZ and SZ designed the study, collected and analyzed data, and drafted the manuscript. GC helped in the statistical analysis and result interpretation. HW, GC, CL, and PY prepared the figures and interpreted the results. YL supervised the study. PY and DK were identified as the guarantor of the paper, taking responsibility for the integrity of the work as a whole. All authors read and approved the final manuscript.

## Funding

This study was supported by Fujian Clinical Research Center for Neurological Disease (SSJ-YJZX-1to DK).

## Conflict of Interest

The authors declare that the research was conducted in the absence of any commercial or financial relationships that could be construed as a potential conflict of interest.

## Publisher's Note

All claims expressed in this article are solely those of the authors and do not necessarily represent those of their affiliated organizations, or those of the publisher, the editors and the reviewers. Any product that may be evaluated in this article, or claim that may be made by its manufacturer, is not guaranteed or endorsed by the publisher.
